# A Mid-Scapular Portal for Arthroscopic-Assisted Fixation of Severe Retraction Greater Tuberosity Avulsion Fracture

**DOI:** 10.1016/j.eats.2022.07.002

**Published:** 2022-10-20

**Authors:** Khananut Jaruwanneechai, Artit Boonrod

**Affiliations:** Department of Orthopaedics, Faculty of Medicine, Khon Kaen University, Khon Kaen, Thailand

## Abstract

Chronic displaced greater tuberosity avulsion fracture of the humerus causes severe retraction of the rotator cuff and resorption of the avulsion fragment. Many treatment options can be considered to solve this problem and return the patient to function. The arthroscopic technique is very challenging to achieve a reduction of the rotator cuff and fixation of greater tuberosity with minimized soft-tissue damage. This Technical Note describes a portal for arthroscopic-assisted reduction and fixation in severe retracted greater tuberosity avulsion fracture. The technique is easy to release and fix chronic displaced greater tuberosity and could avoid unnecessary open surgery.

Most greater tuberosity fractures are nondisplaced or minimally displaced and can be treated with nonoperative treatment.^1^, ^2^, ^3^, ^4^ However, later greater tuberosity displacement was reported at about 50%.^2^ Chronic greater tuberosity avulsion fracture of the humerus is a very rare presentation. It is caused by inappropriate rehabilitation, lost follow-up, or misdiagnosis of nondisplaced greater tuberosity fracture. Ogawa et al.^5^ reported that the greater tuberosity avulsion fracture tends to be posteriorly and superiorly displaced due to retraction of supraspinatus and infraspinatus, causing chronic shoulder pain and functional impairment. The very important problem that is different from acute injury is large displacement and bony resorption of avulsion fragments. Surgery is the treatment of choice for greater tuberosity avulsion fracture with displacement of more than 5 mm.^3^^,^^4^ A nonunion fracture can develop, especially in chronic cases. An avulsion fragment typically develops into an eggshell fracture, resulting in insufficient stability for purchased screw fixation or plate osteosynthesis techniques.^6^^,^^7^

Although open techniques are preferred for large displacement and severe comminution, the arthroscopic technique can enhance visualization to address concomitant injury and preserve the soft tissue around the surgical site. Many arthroscopic techniques have been shown to be used for non- or minimal displacement of greater tuberosity avulsion fractures,^8^, ^9^, ^10^, ^11^, ^12^ but there has been no study of chronic cases with severe retraction. This technique, proposed by author A.B., describes an additional portal at mid-scapula to aid in the reduction and fixation of severe retraction of greater tuberosity avulsion fracture.

## Surgical Technique (With Video Illustration)

**Fig 1:**
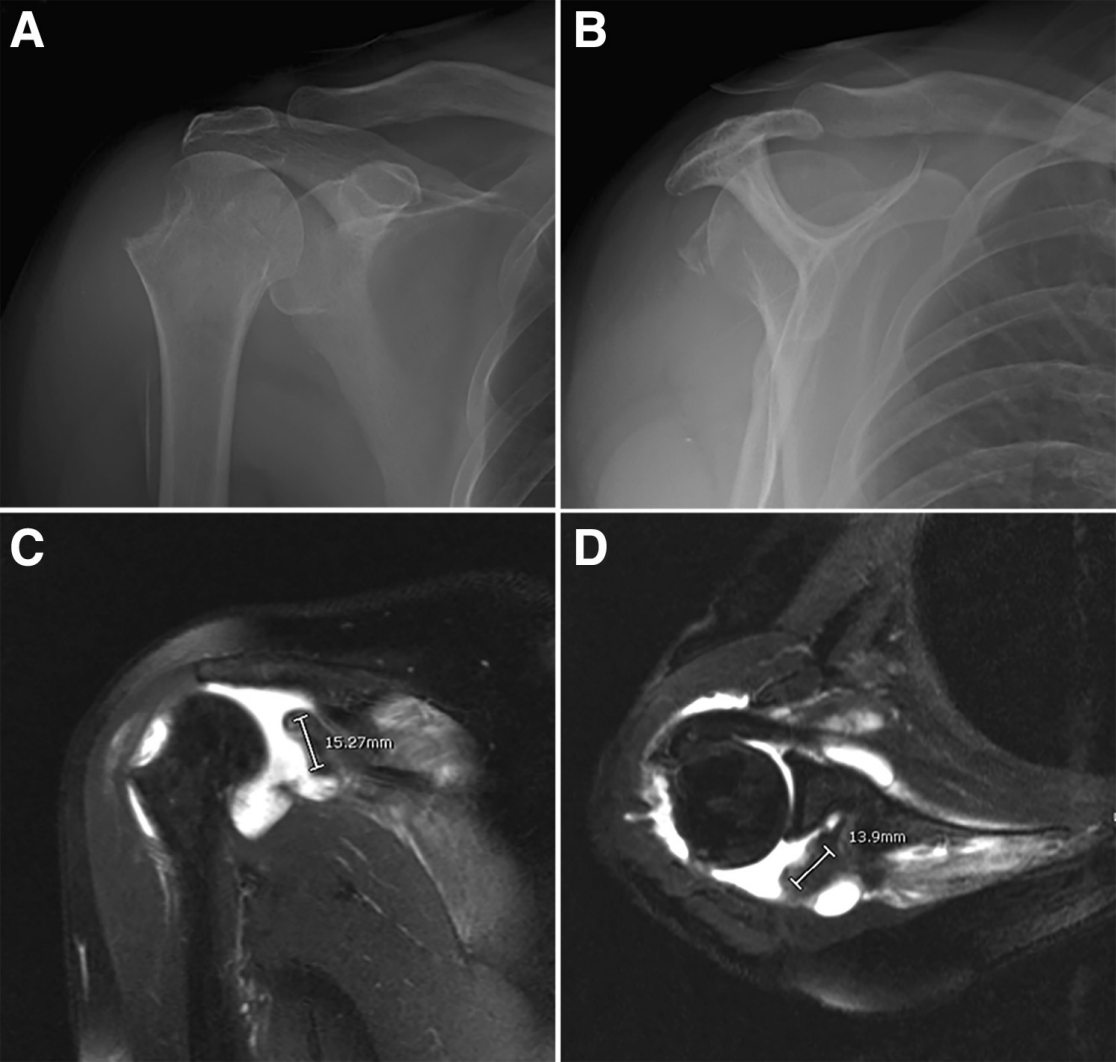
A patient presented with chronic severe retraction of the greater tuberosity avulsion fractured right shoulder. (A) Radiograph, anteroposterior view showed medial displaced greater tuberosity fracture. (B) Radiograph, lateral view showing posterior displaced greater tuberosity fracture. (C, D) Magnetic resonance imaging (1.5 Tesla). (C) Oblique coronal T2-weighted with fat saturation showed medial displacement of avulsion fragment. (D) Axial T2-weighted with fat saturation showed posterior displacement of avulsion fragment.

A preoperative radiograph and magnetic resonance imaging are required to evaluate the fracture pattern, degree of displacement, size of the bony avulsion, and other associated injuries. The supraspinatus and infraspinatus tendons cause a large displaced fragment posterior to the glenoid neck (Fig 1). The patient under general anesthesia is placed in a beach-chair position with a deviated trunk laterally to expose the medial border of the scapular as a landmark for inserting the mid-scapular portal. The authors recommend the beach-chair position because it allows arm manipulation during the procedure, and it is easy to make an orientation. In addition, this position facilitates conversion to an open approach if necessary.

The technique of arthroscopic fixation by using the mid-scapular portal is demonstrated in Video 1. The key concepts for success with this procedure are listed in Table 1. The 30° arthroscope is placed through a standard posterior portal at a soft spot 1 cm medial and 1 cm inferior to the posterolateral edge of the acromion. Any intra-articular lesion is addressed throughout a diagnostic arthroscopy. A subacromial bursectomy is performed using an arthroscopic shaver and radiofrequency device through a lateral portal to enhance visualization. The camera is positioned at the posterolateral portal, which provides good visibility of the fracture fragment and rotator cuff. The size, comminution, and direction of displacement of the greater tuberosity fragment are all closely scrutinized. An arthroscopic shaver can be used to debride the fracture bed to enhance healing potential carefully. The camera is positioned at the posterolateral portal, which provides good visibility of the fracture fragment.

**Table 1:** Pearls and Pitfalls of a Mid-Scapular Portal for Arthroscopic-Assisted Fixation

	Pearls	Pitfalls
Position	To increase the working room in subacromial space, the patient should be in the beach-chair position with the arms hanging or the lateral position with the lateral traction device.	It is difficult to access and repair the greater tuberosity fragment when the arm is positioned without traction (gravity or device)
Draping	The medial border of the scapular should be free and palpable to be a landmark for creating the mid-scapular portal.	It is difficult to locate the mid-scapular portal if the draping is too lateral.
Portals	The spinal needle is always used to check the direction of the mid-scapular portal before creating.	The landmark of the portal is difficult to locate if the subcutaneous tissue is swollen.

**Fig 2:**
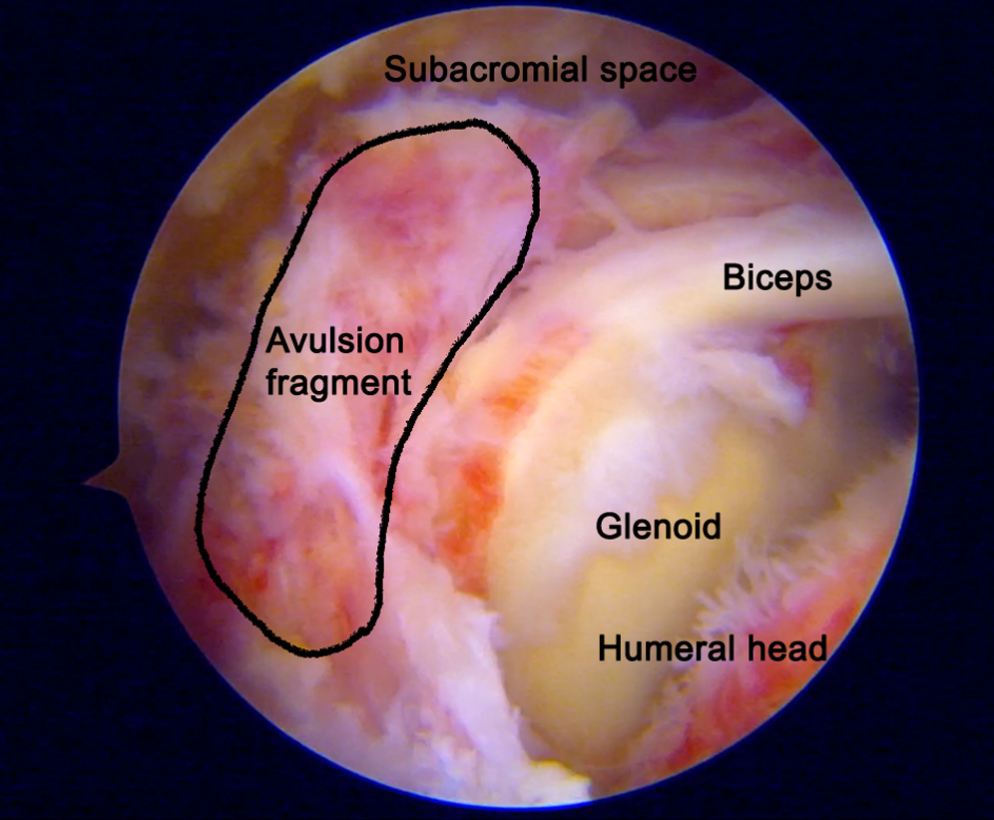
A subacromial view from a posterolateral portal in the beach-chair position showed a greater tuberosity avulsion fragment in the black outline.

**Fig 3:**
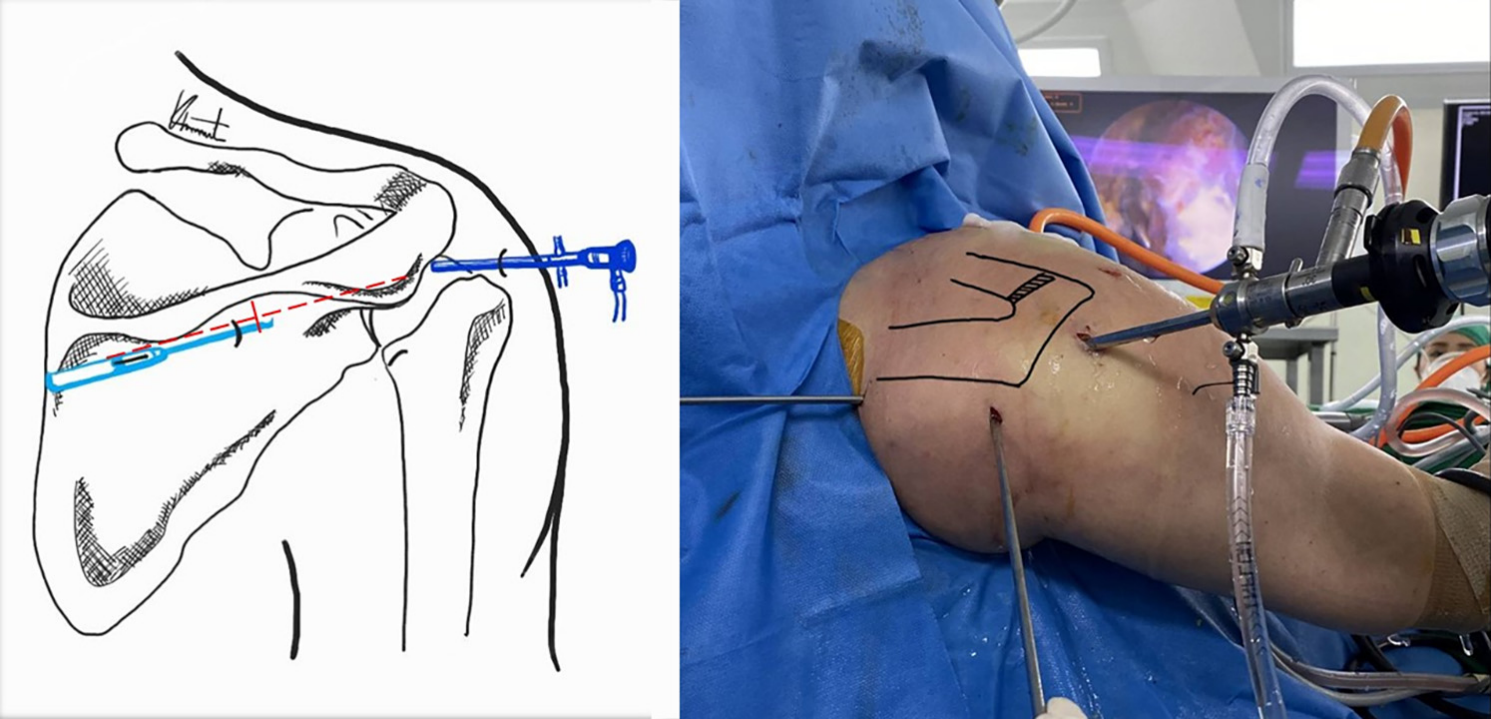
The posterior view of the right shoulder demonstrates the entry point of the mid-scapular portal (arrow). The anatomic landmark for the mid-scapular portal is shown in the graphic. The portal is below the scapular spine and center between the acromion and medial border of the scapula.

**Fig 4:**
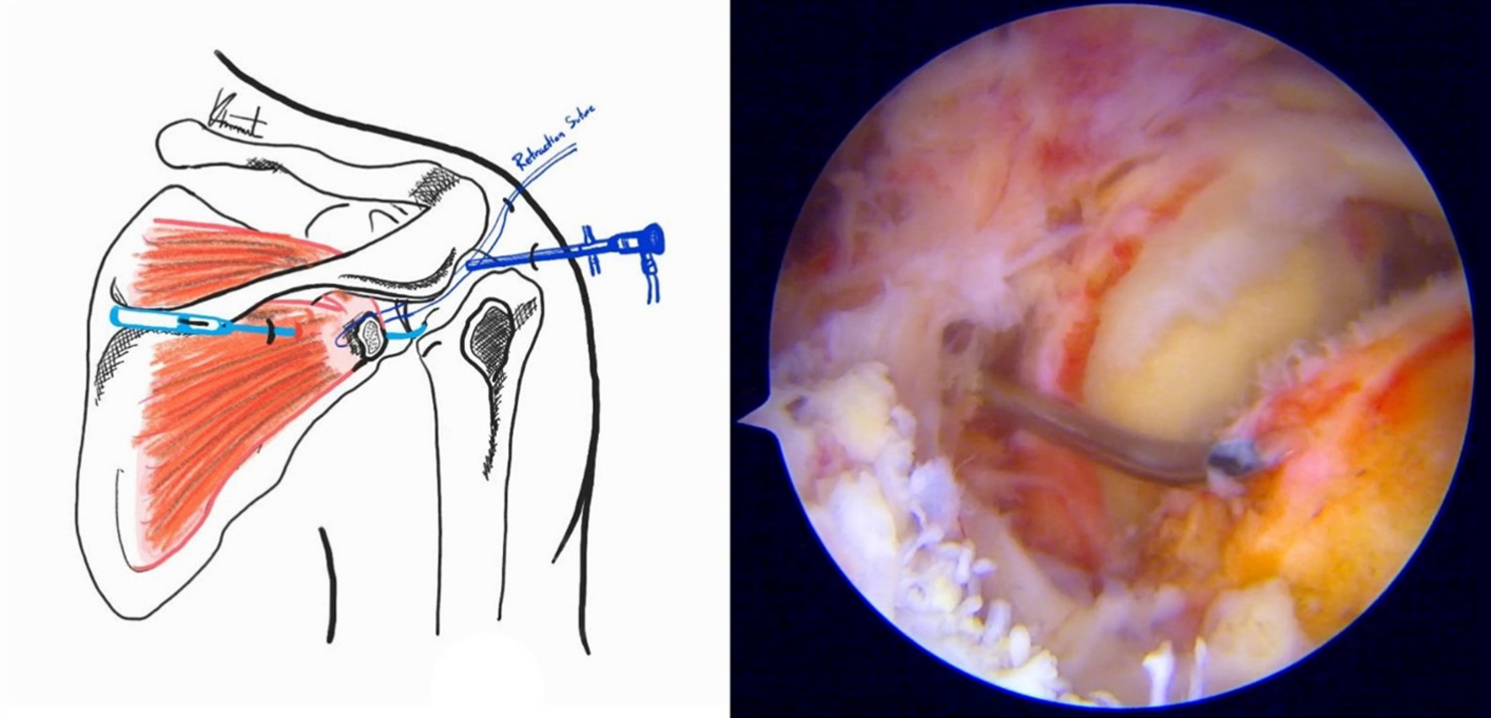
A subacromial view from a posterolateral portal in the beach-chair position demonstrated repairing avulsion fragments by a suture passing device. The device is passed through the mid-scapular portal to assist in the reduction of the avulsion fragment.

**Fig 5:**
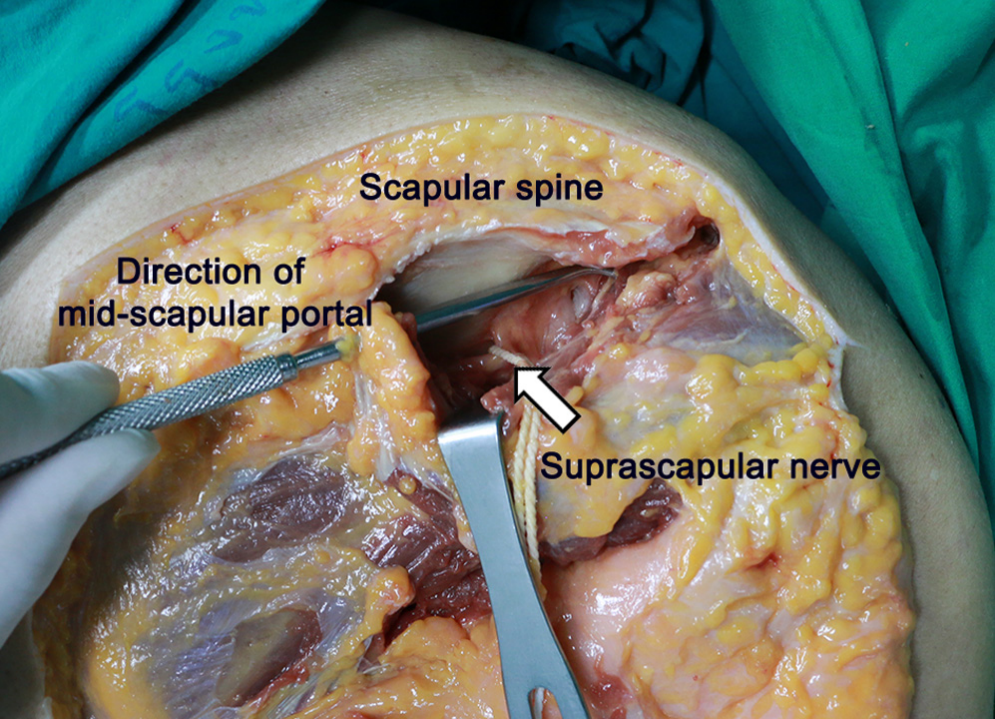
The posterior view of the right shoulder in the cadaveric dissection demonstrated that the instrument inserted from the mid-scapular portal should stay close to the scapular spine to avoid iatrogenic injury to the suprascapular nerve (arrow).

The fracture site and avulsion fragment are visualized in Fig 2. Supraspinatus tendon and infraspinatus tendons pull the bony fragment in a posterosuperior direction. An anterolateral working portal is established to apply the traction suture if it is difficult to reduce the avulsion fragment. The mid-scapular portal is created, which a landmark is in the middle of the scapular in the mediolateral plane and just inferior to the scapular spine. The traction suture is passed from the mid-scapular portal through the rotator cuff tissue via the suture passing device (SutureLasso SD 45°, Curve Right; Arthrex, Naples, FL) as in Fig 3, Fig 4, and Video 1. The course of the suprascapular nerve must be kept in mind. It passes through the suprascapular notch and under the transverse scapular ligament to innervate the supraspinatus muscle. The instrument should be placed close to the scapular spine to avoid injury to the suprascapular nerve (Fig 5). The process is then repeated for more traction sutures to assist in the reduction and extensively release the tendon adhesion. Before fracture fixation, the fracture fragment is mobilized to ensure that it can be reduced anatomically. An arthroscopic shaver and curette can be used to carefully debride the fracture bed at the proximal humerus to enhance healing potential.

**Fig 6:**
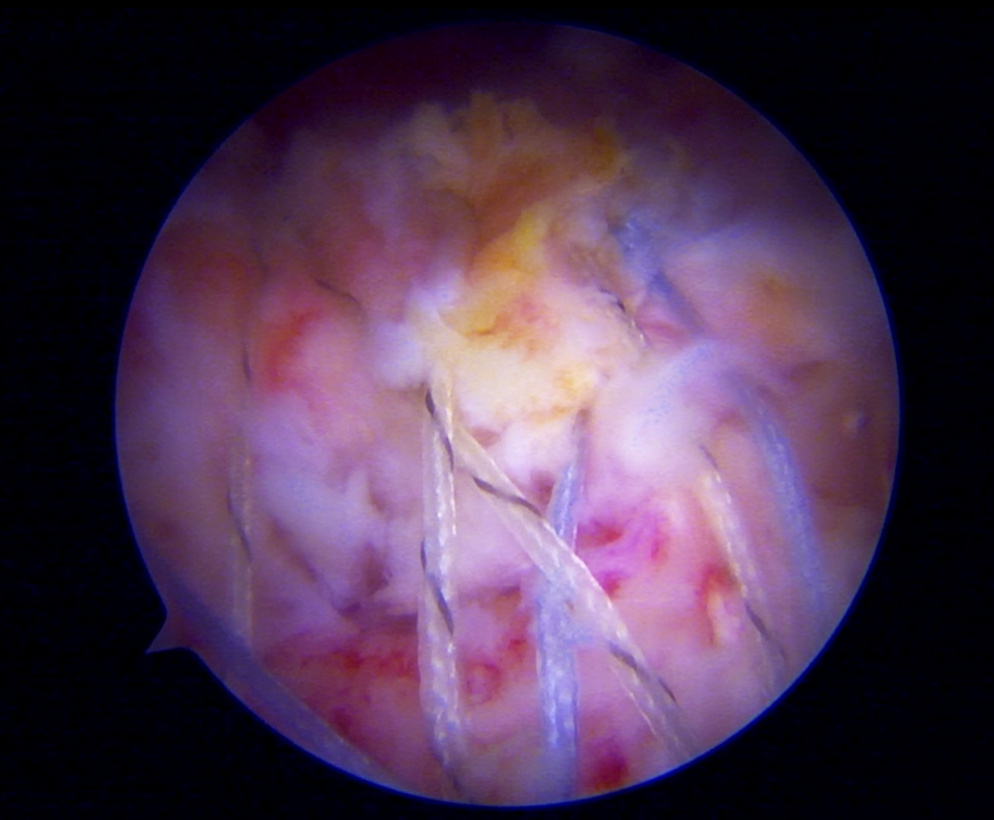
A subacromial view from a posterolateral portal in the beach-chair position showed the greater tuberosity repaired with double-row suture bridge fixation.

Due to the fragment’s size and resorption concerns, the fracture should be treated with a double-row construct with suture anchors similar to those for a massive rotator cuff tear. Because of greater tuberosity bone loss around the fracture site, anchor placement may be difficult, and anchor pullout may occur if the anchors are placed within the fracture bed. The secure medial row anchors (BioComposite Corkscrew FT Suture Anchor, 5.5 mm × 14.7 mm; Arthrex) should be inserted into the intact cortical bone at the fracture edge. The anchor’s trajectory should be away from the fracture site. The camera is usually placed in the lateral portal during suture passage and fracture reduction. With the assistance of an arthroscopic grasper, a suture shuttling device is used to pass the suture from the medial row anchor through the rotator cuff tendon. Depending on the fracture configuration, sutures can be loaded through either the anterior or posterior portal. Medial row sutures are subsequently tied after adequate reduction has been achieved. After all, both suture strands are threaded through the knotless lateral row fixation (BioComposite SwiveLock C, 4.75 mm ×19.1 mm; Arthrex). This construction compresses the rotator cuff laterally into an anatomic position while simultaneously reducing the displaced greater tuberosity fracture. The position of the fracture fragment and the restoration of the rotator cuff tendon should reveal a near-anatomic reduction after the surgery (Fig 6). Fluoroscopic imaging and gentle motion should demonstrate stable fixation under direct visualization.

The patient remains immobilized in an abduction sling during the early postoperative period, focusing on passive motion exercise while allowing elbow and hand motion. At 6 weeks, physical therapy is initiated for active assisted motion, while strengthening exercises are started at 12 weeks postoperatively.

## Discussion

Surgical considerations for greater tuberosity fractures have been described in various publications, with the objective of surgical treatment to restore normal anatomy and allow early motion with secure fixation. Numerous fixation techniques have been proposed for greater tuberosity fractures. The size of the fragment, degree of displacement, degree of comminution, and the presence of osteoporosis all affect the selection of an appropriate surgical method. In this technique, authors use an additional portal called the “mid-scapular portal” to reduce the greater tuberosity fragment in chronic severe retraction. There are advantages to this arthroscopic technique and some limitations, as in Table 2.

**Table 2:** Advantages and Disadvantages of a Mid-Scapular Portal for Arthroscopic-Assisted Fixation

Advantages	Disadvantages/Risks
Need only an additional portal from standard portals for reducing and fixing Optimal visualization and reduction Treatment of any relevant lesion Minimally invasive procedure Quicker recovery	Requires advanced shoulder arthroscopic skills Suprascapular and axillary nerve injury Requires special equipment Longer operative time Greater procedure cost

Recently, with the improvement of arthroscopic surgery, arthroscopic reduction and fixation of greater tuberosity fractures have been accomplished. In addition, suture anchor fixation is suitable for comminuted greater tuberosity fractures. It increases implant purchase and decreases the chance of future fracture fragment comminution compared to other fixation techniques. Numerous shoulder surgeons have recently used the double-row repair technique, and a substantial biomechanical study on this rotator cuff repair technique has been published. Several studies report 80% to 90% good-to-excellent outcomes of using the arthroscopic double-row suture anchor fixation technique for displaced comminuted greater tuberosity fractures with minimal complications such as shoulder stiffness, anchor protrusion, and impingement.^8^^,^^9^^,^^13^, ^14^, ^15^ In 2016, Liao et al.^16^ published a study of 32 patients with displaced greater tuberosity avulsion fracture comparing arthroscopic with open surgery. The arthroscopic group had a longer operative time but had a greater range of motion and functional scores, and 3 patients who underwent open surgery required revision surgery. The disadvantages of arthroscopic surgery are the steeper learning curve for the surgeon and the longer operating time required in comparison with open surgery. The advantages of arthroscopic fixation of greater tuberosity fractures are that it is less invasive, can identify and treat concurrent pathologies such as labral and rotator cuff tears, and reduces complications from open surgery such as implant irritation and nerve injury.

However, the fracture displacement in a recent study was up to 1 cm. The patient underwent surgery within 1 month. Given the lack of literature dealing with the management of chronic and large displacement of a greater tuberosity avulsion fracture, almost all studies suggest that open surgery should probably be considered.^10^ The bony fragment often has been resorbed in a chronic fracture and is also challenging to reduce due to soft-tissue contracture. The chronic medial retraction of the rotator cuff could cause fixation failure due to high tension after fixation.^17^

The authors propose the mid-scapular portal, a modification of the accessory posteromedial portal described by Glenn et al.,^18^ to assist suture passing through the infraspinatus during arthroscopic reduction and fixation of the greater tuberosity. It minimizes the need for curved or angled suture relay devices and assists reduction through the retracted tendon while preserving bony fragments. This simple method allows for reduction and fixation of the severed displacement greater tuberosity avulsion fracture with favorable results. The essential advantage of this technique is that it is easy to release and fix chronic severe, displaced greater tuberosity without open surgery. The crucial risk of this technique is suprascapular nerve injury. To prevent injury, the instrument inserted from the mid-scapular portal should stay close to the scapular spine.

## Supplementary Data

ICMJE author disclosure forms

Video 1 This technical video demonstrates the use of additional mid-scapular portal to facilitate repairing of chronic severe retraction of greater tuberosity avulsion fracture. The patient was prepped and draped in a beach-chair position. Arthroscopic release and repair of the retracted bony fragment were performed using standard arthroscopic and additional mid-scapular portals. The avulsion fragment was repaired to the anatomical location with a double-row suture bridge repair construct.
